# Supplementary material for: Actinide-lanthanide single electron metal-metal bond formed in mixed-valence di-metallofullerenes
Source: Nat Commun. 2023 Oct 20;14:6637. doi: 10.1038/s41467-023-42165-x (PMC10589252; doi:10.1038/s41467-023-42165-x)
Supplement: Supplementary file 1 — Supplementary Information [file 41467_2023_42165_MOESM1_ESM.pdf]

Supplementary Information

Actinide-Lanthanide Single Electron Metal-Metal Bond Formed in Mixed-Valence Di-metallofullerenes

Yingjing Yan,[1†] Laura Abella,[2†] Rong Sun,[3†] Yu-hui Fang,[3] Yannick Roselló,[2] Yi Shen,[1] Meihe Jin,[1] Antonio Rodríguez-Fortea,[2] Coen de Graaf,[2,4] Qingyu Meng,[1] Yang-Rong Yao,[1, 5]* Luis Echegoyen,[6] Bing-Wu Wang,[3]* Song Gao,[3] Josep M. Poblet,[2]* and Ning Chen[1]*

Affiliations:

[1] College of Chemistry, Chemical Engineering and Materials Science, and State Key Laboratory of Radiation Medicine and Protection, Soochow University, Suzhou, Jiangsu 215123, P. R. China

[2] Departament de Química Física i Inorgànica. Universitat Rovira i Virgili, Marcel·lí Domingo 1, 43007 Tarragona, Spain

[3]Beijing National Laboratory for Molecular Sciences, State Key Laboratory of Rare Earth Material Chemistry and Application, College of Chemistry and Molecular Engineering, Peking University, Beijing 100871, P. R. China

[4] ICREA, Pg. Lluís Companys 23, 08010 Barcelona, Spain

[5] Department of Materials Science and Engineering, University of Science and Technology of China, Hefei, 230026, P. R. China

[6] Department of Chemistry, University of Texas at El Paso, 500 W University Avenue, El Paso, Texas 79968, United States

[†]These authors contributed equally: Yingjing Yan, Laura Abella, Rong Sun

*Correspondence authors: chenning@suda.edu.cn; josepmaria.poblet@urv.cat; wangbw@pku.edu.cn; yryao@ustc.edu.cn

Supplementary Figures

Fig.1. HPLC chromatogram and mass spectrum of purified ThDy@C_{2n} (2*n* =72, 76, 78, and 80) and ThY@C_{2n} (2*n* = 72 and 78)...S6

Fig.2. The first separating step of ThDy@C_{72}, ThDy@C_{76}, ThDy@C_{78}, and ThDy@C_{80} S7

Fig.3. The multistage HPLC separation processes of ThDy@C_{72} S8

Fig.4. The multistage HPLC separation processes of ThDy@C_{78} S9

Fig.5. The multistage HPLC separation processes of ThDy@C_{76} and ThDy@C_{80} ..S10

Fig.6. The first separating step of ThY@C_{72} and ThY@C_{78}....................................S11

Fig.7. The multistage HPLC separation processes of ThY@C_{72}. S12

Fig.8. The multistage HPLC separation processes of ThY@C_{78}. S12

Fig.9. Ball and stick representation of disordered metal sites in ThDy@D_2(10611)-C_{72}, ThDy@C_s(17490)-C_{76}, ThDy@D_{3h}(5)-C_{78}, ThDy@I_h(7)-C_{80}, ThY@D_2(10611)-C_{72} and ThY@D_{3h}(5)-C_{78}..S13

Fig.10. DFT-optimized spin-septet ground-state (GS) geometries of ThDy@D_2(10611)-C_{72}, ThDy@C_s(17490)-C_{76}, ThDy@D_{3h}(5)-C_{78} and ThDy@I_h(7)-C_{80}, and spin-doublet GS structures of ThY@D_2(10611)-C_{72} and ThY@D_{3h}(5)-C_{78}. S14

Fig.11. Electronic structure of ThDy@D_{3h}(5)-C_{78}..S15

Fig.12. Molecular orbital (MO) diagram for the ground spin-septet state of ThDy@D_2(10611)-C_{72}, ThDy@C_s(17490)-C_{72}, ThDy@D_{3h}(5)-C_{78}, and ThDy@I_h(7)-C_{80}, and the ground spin-doublet state of ThY@D_2(10611)-C_{72} and ThY@D_{3h}(5)-C_{78}. S15

Fig.13. Spin density distribution with an isosurface of ±0.002 au for ThDy@D_2(10611)-C_{72}, ThDy@C_s(17490)-C_{76}, ThDy@D_{3h}(5)-C_{78}, ThDy@I_h(7)-C_{80}, ThY@D_2(10611)-C_{72} and ThY@D_{3h}(5)-C_{78}...S16

Fig.14. Electronic structure properties for ThDy@C_s(17490)-C_{76}...........................S16

Fig.15. Isosurface (±0.030 au) of the molecular orbitals (MOs) for the σ bonding and Dy f^9 of ThDy@D_2(10611)-C_{72} S17

Fig.16. Isosurface (±0.030 au) of the molecular orbitals (MOs) for the σ bonding and Dy f^9 of ThDy@C_s(17490)-C_{76}....................S17
Fig.17. Isosurface (±0.030 au) of the molecular orbitals (MOs) for the σ bonding and Dy f^9 of ThDy@D_{3h}(5)-C_{78}....................S18
Fig.18. Isosurface (±0.030 au) of the molecular orbitals (MOs) for the σ bonding and Dy f^9 of ThDy@I_h(7)-C_{80}....................S18
Fig.19. Localized (Boys-Foster) molecular orbitals (LMOs) for ThDy@D_{3h}(5)-C_{78}. S19
Fig.20. Calculated EPR spectrum of ThY@D_{3h}(5)-C_{78}....................S19
Fig.21. Isosurface (±0.03 a.u.) of the RAS2 molecular orbitals (MOs) for the spin-septet ground state of ThDy@D_{3h}(5)-C_{78}....................S20
Fig.22. Representation of the molecular orbital diagrams for the neutral, oxidation and reduced forms of ThY@D_2(10611)-C_{72}....................S20
Fig.23. Representation of the molecular orbital diagrams for the neutral, oxidation and reduced forms of ThY@D_{3h}(5)-C_{78}. S21
Fig.24. Cyclic voltammogram of ThY@D_2(10611)-C_{72}, ThY@D_{3h}(5)-C_{78}, ThDy@D_2(10611)-C_{72}, ThDy@D_{3h}(5)-C_{78} and ThDy@I_h(7)-C_{80}....................S22
Fig.25. UV-vis−NIR absorption spectra of purified ThDy@C_{2n} ($2n$ = 72, 76, 78, and 80) and ThY@C_{2n} ($2n$ = 72 and 78) S24
Fig.26. ORTEP-style illustration with probability ellipsoids for ThDy@D_2(10611)-C_{72}, ThDy@C_s(17490)-C_{76}, ThDy@D_{3h}(5)-C_{78}, ThDy@I_h(7)-C_{80}, ThY@D_2(10611)-C_{72}, and, ThY@D_{3h}(5)-C_{78}....................S25
Fig.27. Experimental temperature dependence of χ for ThDy@D_{3h}(5)-C_{78}....................S26

Supplementary Tables

Table 1. The cage disorder and the shortest Ni-to-Cage distance of $ThX@C_{2n}$·[Ni^{II}(OEP)] (X=Dy and Y, 2*n* = 72, 76, 78, and 80). S27

Table 2. The fractional occupancies of the metal positions in ThDy@D_2(10611)-C_{72}, ThDy@C_s(17490)-C_{76}, ThDy@D_{3h}(5)-C_{78}, ThDy@I_h(7)-C_{80}, ThY@D_2(10611)-C_{72} and ThY@D_{3h}(5)-C_{78}. S27

Table 3. Closest metal-to-cage contacts in ThDy@D_2(10611)-C_{72}, ThDy@C_s(17490)-C_{76}, ThDy@D_{3h}(5)-C_{78} and ThDy@I_h(7)-C_{80}. S28

Table 4. Closest metal-to-cage contacts in ThY@D_2(10611)-C_{72} and ThY@D_{3h}(5)-C_{78} S29

Table 5. Crystallographic data of ThDy@D_2(10611)-C_{72}/Ni^{II}(OEP), ThDy@C_s(17490)-C_{76}/Ni^{II}(OEP), ThDy@D_{3h}(5)-C_{78}/Ni^{II}(OEP) and ThDy@I_h(7)-C_{80}/Ni^{II}(OEP). S30

Table 6. Crystallographic data of ThY@D_2(10611)-C_{72}/Ni^{II}(OEP) and ThY@D_{3h}(5)-C_{78}/Ni^{II}(OEP) S31

Table 7. Relative energies (in kcal·mol^{-1}) of the RASSCF calculations for the different spin states, and lowest-energy states for ThDy@D_{3h}(5)-C_{78}. S32

Table 8. Relative energies (in kcal·mol^{-1}) and spin distributions of active orbitals with the corresponding weight of the dominant electronic configuration (in %) for the lowest states determined from CASSCF calculations for ThDy@D_{3h}(5)-C_{78}. S33

Table 9. Redox potentials (V vs. Fc/Fc$^+$) and electrochemical band gaps of ThY@D_2(10611)-C_{72}, ThY@D_{3h}(5)-C_{78}, ThDy@D_2(10611)-C_{72}, ThDy@D_{3h}(5)-C_{78}, ThDy@I_h(7)-C_{80} and the selected reference EMFs. S33

Table 10. Experimental and calculated first oxidation and reduction potentials (in eV) for ThY@C_{2n} (2*n* = 72 and 78) S34

Table 11. Relative energies (in kcal·mol^{-1}) for the singlet and triplet spin states for the oxidation and reduction of ThY@C_{2n} (2*n* = 72 and 78) S34

Table 12. The HPLC retention time and details of the UV-vis-NIR absorptions of ThDy@C_{2n} (2*n* = 72, 76, 78, and 80) and ThY@C_{2n} (2*n* = 72 and 78) S34

Table 13. Selected distances compared between ThDy@D_{3h}(5)-C_{78} and Dy_2@C_{79}N/ Dy_2@C_{80}(CH_2Ph)........S35
References........S36

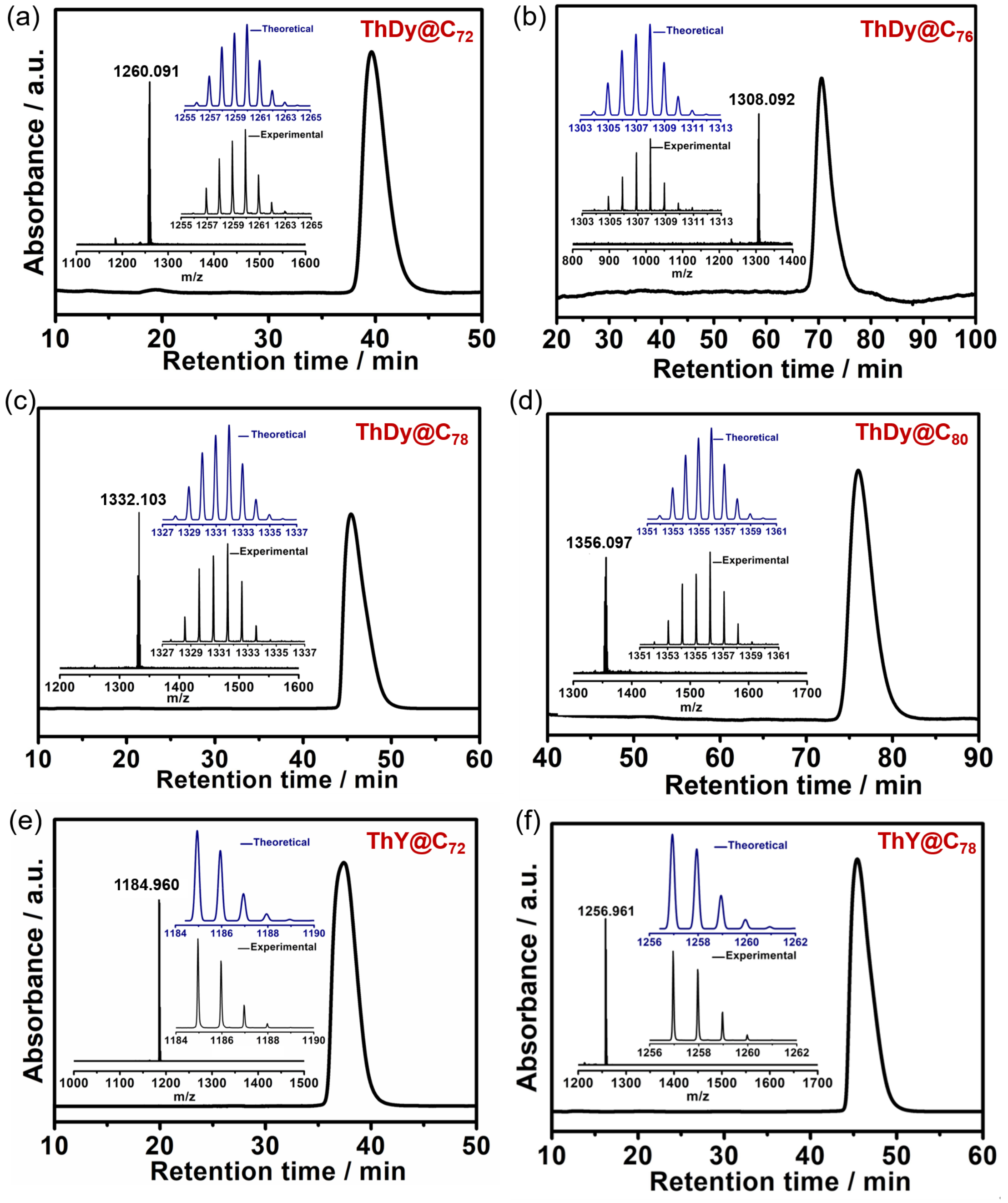

Supplementary Fig.1. HPLC chromatogram of purified (a-d) ThDy@C_{2n} ($2n$ = 72, 76, 78, and 80) and (e-f) ThY@C_{2n} ($2n$ = 72 and 78) on a Buckyprep column with toluene as the eluent. HPLC conditions: λ = 310 nm; flow rate = 4 mL/min. The insets show the positive-ion mode MALDI-TOF mass spectra and expansions of the corresponding experimental isotopic distribution of the compound in comparison with the calculated one.

HPLC separation of ThDy@C_{72}, ThDy@C_{76}, ThDy@C_{78} and ThDy@C_{80}. The first stage was performed on a Buckyprep-M column (25 × 250 mm, Cosmosil Nacalai Tesque) with toluene as mobile phase. The fraction of 28.5-30.5 min (marked in red) is collected and named L. The fraction of 32.5-34.5 min (marked in blue) is collected and named M. The fraction of 41.5-46 min (marked in dark cyan) is collected and named N. Fraction L and M contain ThDy@C_{72} and ThDy@C_{78}, respectively. And Fraction N contains ThDy@C_{76} and ThDy@C_{80}. Then the target fractions (L, M, and N) were enriched and injected into Buckyprep column for next stage separation.

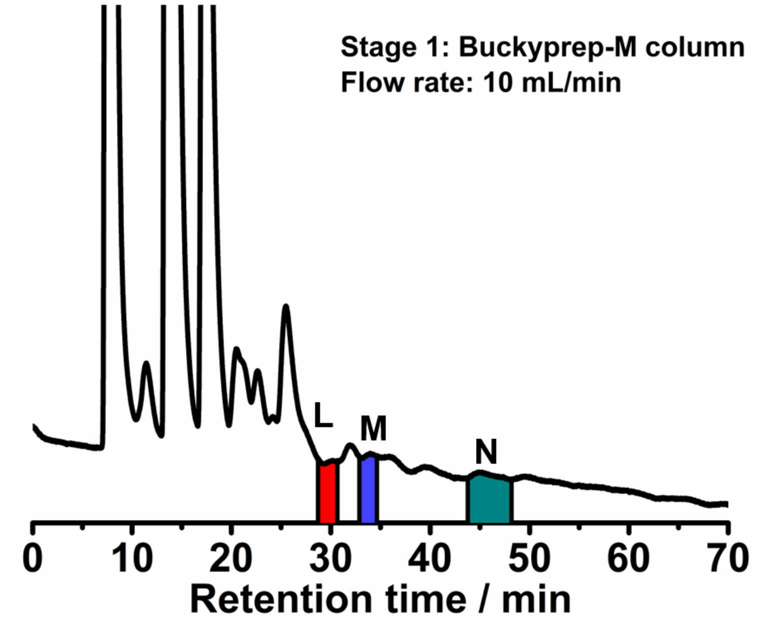

Supplementary Fig.2. The first separating step of ThDy@C_{72}, ThDy@C_{76}, ThDy@C_{78}, and ThDy@C_{80}.

HPLC separation of ThDy@C_{72}. The fraction L was injected into Buckyprep column to collect eluent from 34 to 42 min (L-1). Then Fraction L-1 was carried out on the Buckyprep-M column with toluene as the mobile phase to obtain L-1-1. After that, the L-1-1 was recycled on the Buckyprep column for purification. Finally, the pure sample of ThDy@C_{72} was obtained at m/z = 1260.091.

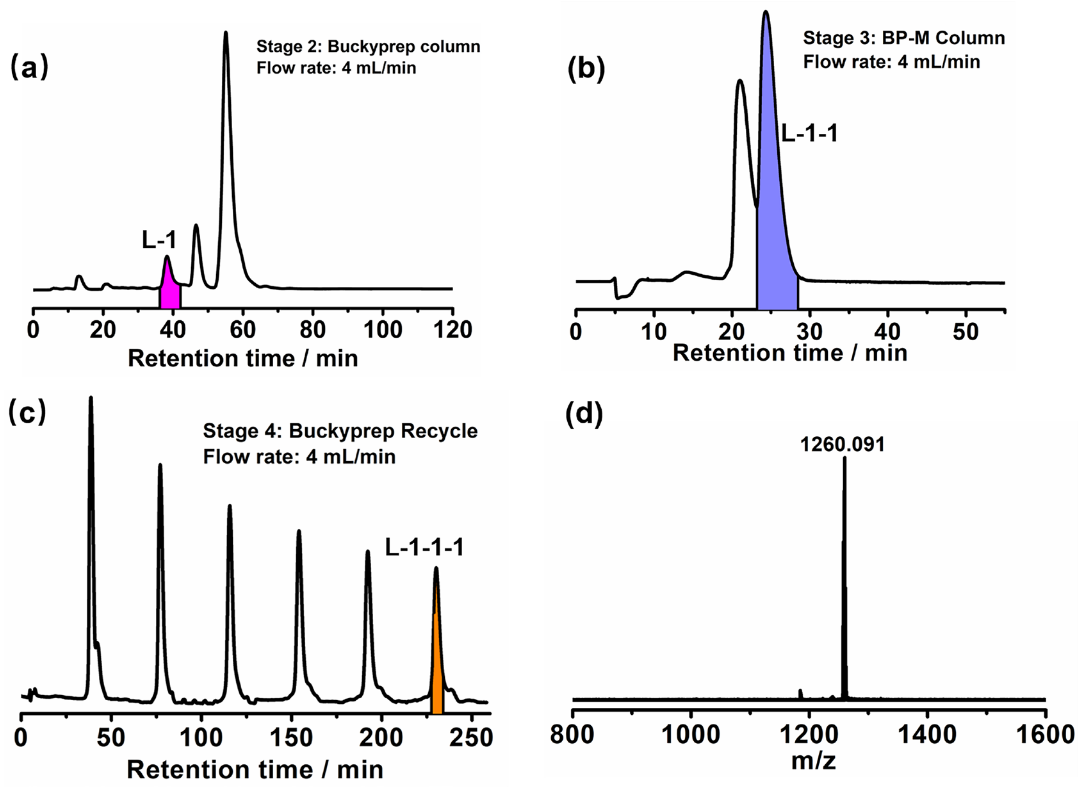

Supplementary Fig.3. The multistage HPLC separation processes of ThDy@C_{72}. (a) The second separating step of ThDy@C_{72}. (b) The third separating step of ThDy@C_{72}. (c) The fourth separating step of ThDy@C_{72}. (d) The pure sample's MALDI-TOF mass spectrum.

HPLC separation of ThDy@C_{78}. The fraction M marked in blue was injected into Buckyprep column for next separation. The eluent from 42-48.5 min (M-1) was collected which contains the target product ThDy@C_{78}. After separation from 5PBB column, the purified sample ThDy@C_{78} was obtained along with the single peak of MS (see Fig.4c).

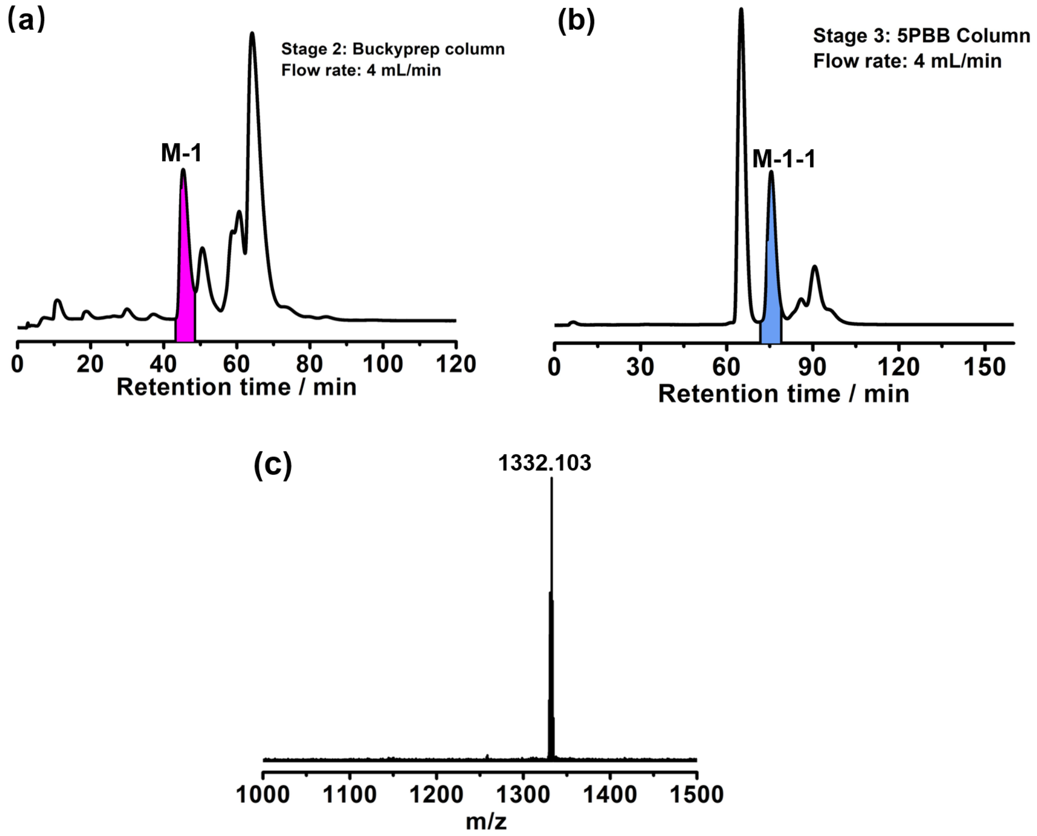

Supplementary Fig.4. The multistage HPLC separation processes of ThDy@C_{78}. (a) The second separating step of ThDy@C_{78}. (b) The third separating step of ThDy@C_{78}. (c) The pure sample's MALDI-TOF mass spectrum.

HPLC separation of ThDy@C_{76} and ThDy@C_{80}. The faction N was enriched. After concentrating, fraction N was injected into Buckyprep column (10 × 250 mm, Cosmosil Nacalai Tesque), eluent from 66-74 min marked N-1 (lake blue) and 74-82 min (N-2) marked in (magenta) was collected, which contains ThDy@C_{76} and ThDy@C_{80}, respectively. Then N-1 was carried out on the 5PBB column, as a result, the relatively pure sample ThDy@C_{76} (N-1-1) was obtained along with single peak MS at m/z = 1308.092 (as shown in Fig.5f).

Similarly, the faction N-2 was performed on 5PBB column and the eluent from 82-95 min was collected. After further recycling purified on Buckyprep-M column, the pure sample ThDy@C_{80} marked in orange was obtained at m/z = 1356.097 (see Fig.5f).

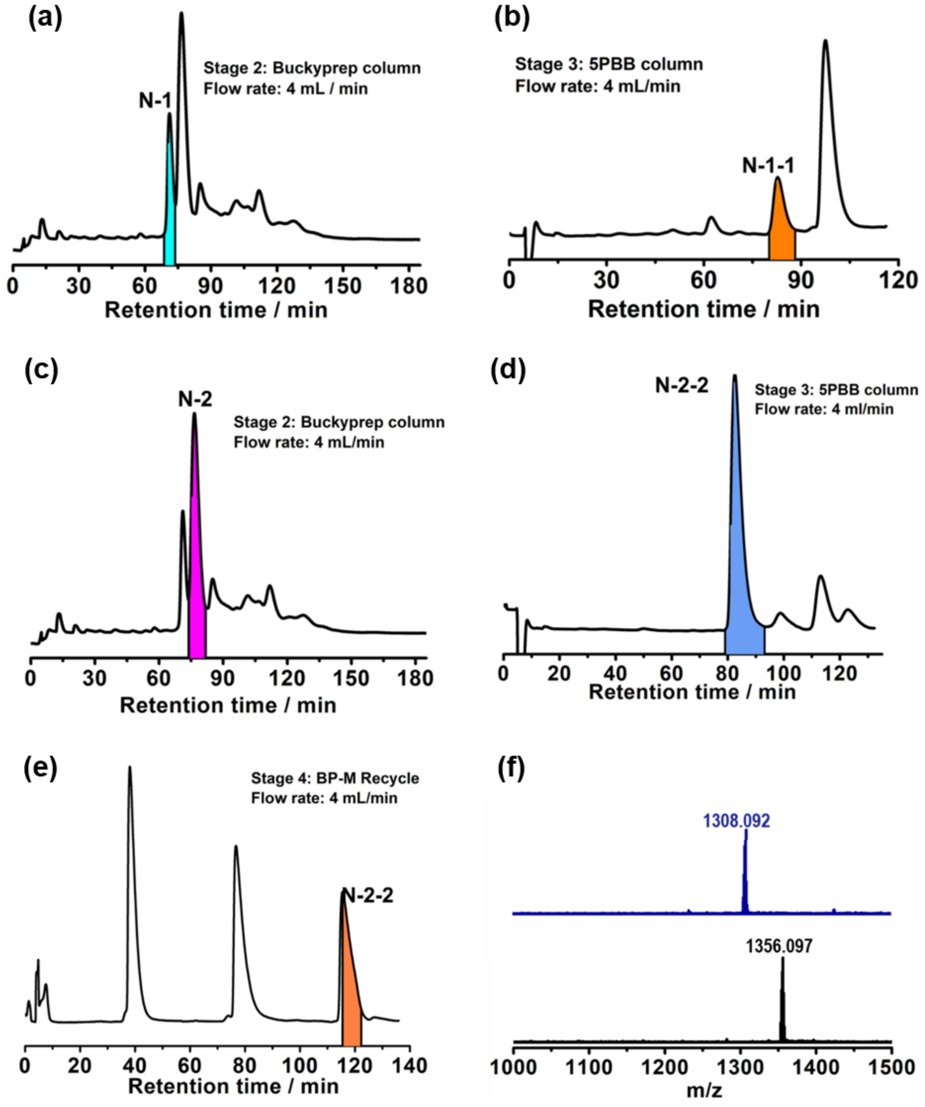

Supplementary Fig.5. The multistage HPLC separation processes of ThDy@C_{76} and ThDy@C_{80}. (a) The second separating step of ThDy@C_{76}. (b) The third separating step of ThDy@C_{76}. (c) The second separating step of ThDy@C_{80}. (d) The third separating step of ThDy@C_{80}. (e) The fourth separating step of ThDy@C_{80}. (f) The pure sample's MALDI-TOF mass spectrum.

HPLC separation of ThY@C_{72} and ThY@C_{78}. The first stage was performed on a Buckyprep-M column (25 × 250 mm, Cosmosil Nacalai Tesque) with toluene as mobile phase. Then collected the fractions of 28.5-30.5 min and 32.5-34 min and marked them as A and B, which contain ThY@C_{72} and ThY@C_{78}, respectively.

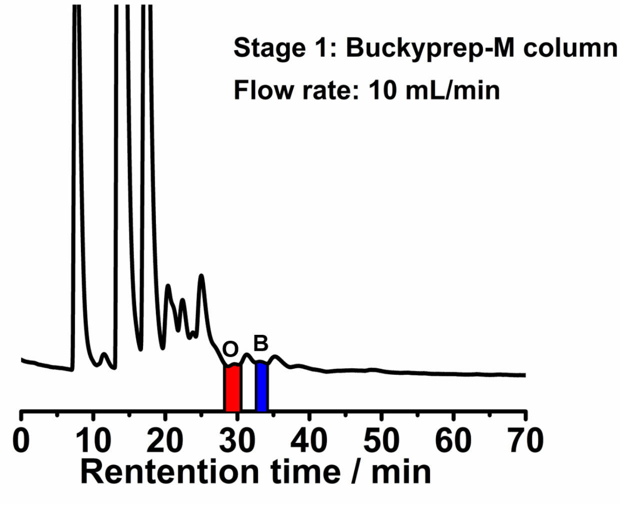

Supplementary Fig.6. The first separating step of ThY@C_{72} and ThY@C_{78}.

HPLC profiles of ThY@D_2(10611)-C_{72}. After concentrating, fraction O was injected into Buckyprep column (10 × 250 mm, Cosmosil Nacalai Tesque) and eluent from 35-42 min marked O-1(magenta) was concentrated and performed on Buckyprep-M column for next stage separation. After removing most of the hollow cage C_{84}, the fraction retention time at 22-26 min Buckyprep-M column O-1-1 was obtained. Then O-1-1 is injected into 5PBB column to purify. Finally, the target fraction of 48-55 min from 5PBB column was purified and confirmed by single peak of the MALDI-TOF mass spectrometry (see Fig.7d).

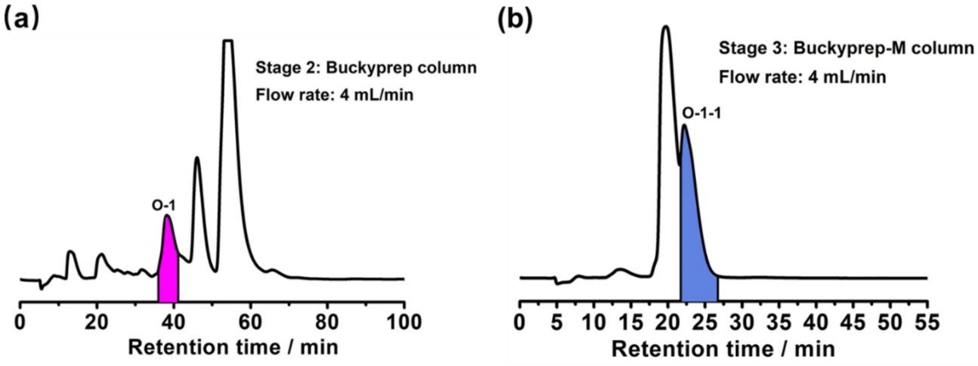

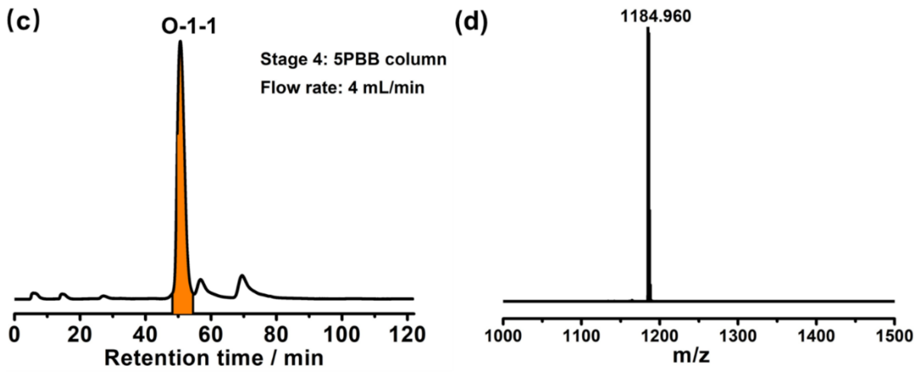

Supplementary Fig.7. The multistage HPLC separation processes of ThY@C_{72}. (a) The second separating step of ThY@C_{72}. (b) The third separating step of ThY@C_{72}. (c) The fourth separating step of ThY@C_{72}. (d) The pure sample's MALDI-TOF mass spectrum.

HPLC profiles of ThY@C_{78}. Fraction B was also injected into Buckyprep column (10 × 250 mm, Cosmosil Nacalai Tesque), then the eluent from 42-48.5 min marked as B-1 was collected for enriching ThY@C_{78}. After that, B-1 was injected into 5PBB column (10 × 250 mm, Cosmosil Nacalai Tesque) which performed well on the cage size sequence. As a result, the pure sample of ThY@C_{78} was obtained as B-1-1, along with the MALDI-TOF mass spectrometry in a positively charged mode.

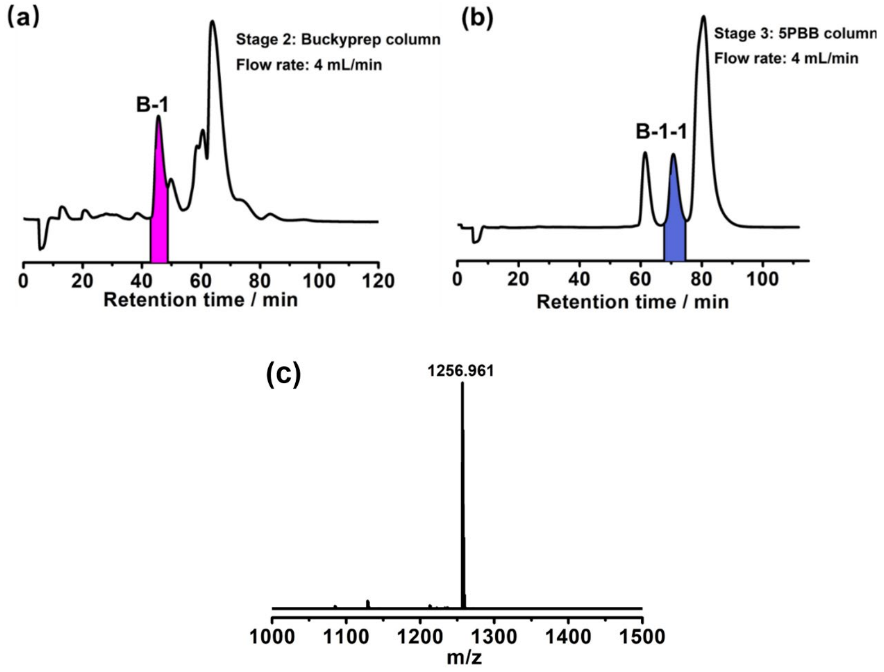

Supplementary Fig.8. The multistage HPLC separation processes of ThY@C_{78}. (a) The second separating step of ThY@C_{78}. (b) The third separating step of ThY@C_{78}. (c) The pure sample's MALDI-TOF mass spectrum.

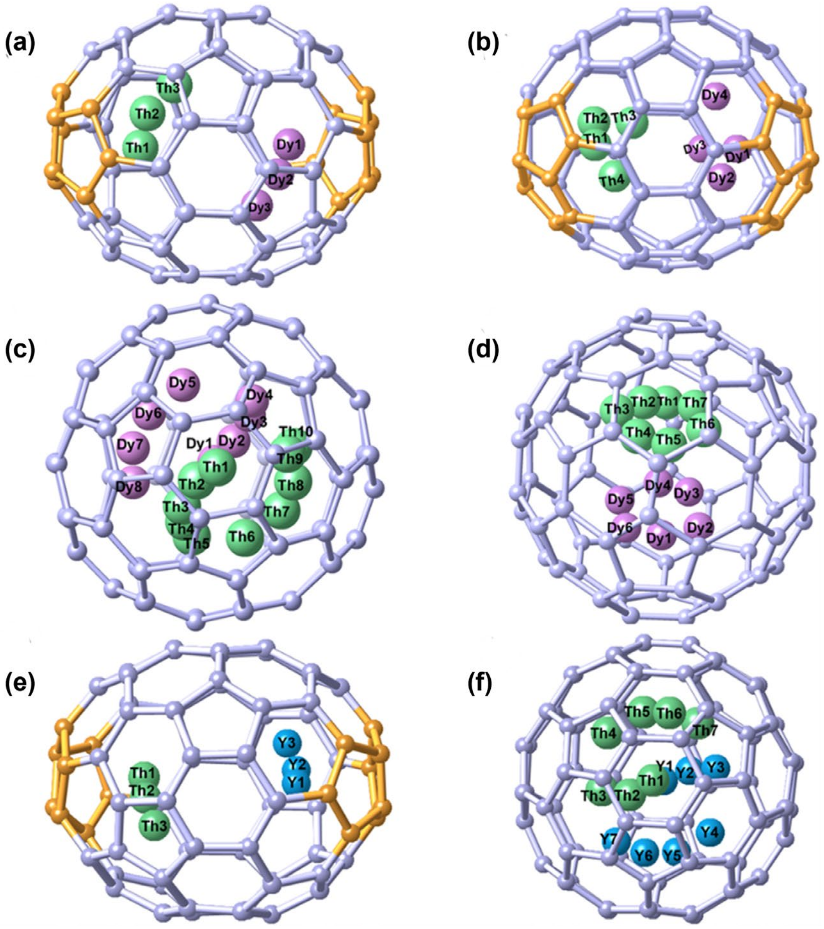

Supplementary Fig.9. Ball and stick representation of disordered metal atom sites in (a) ThDy@D_2(10611)-C_{72}, (b) ThDy@C_s(17490)-C_{76}, (c) ThDy@D_{3h}(5)-C_{78}, (d) ThDy@I_h(7)-C_{80}, (e) ThY@D_2(10611)-C_{72}, and (f) ThY@D_{3h}(5)-C_{78}. For clarity, the major cage orientations are shown.

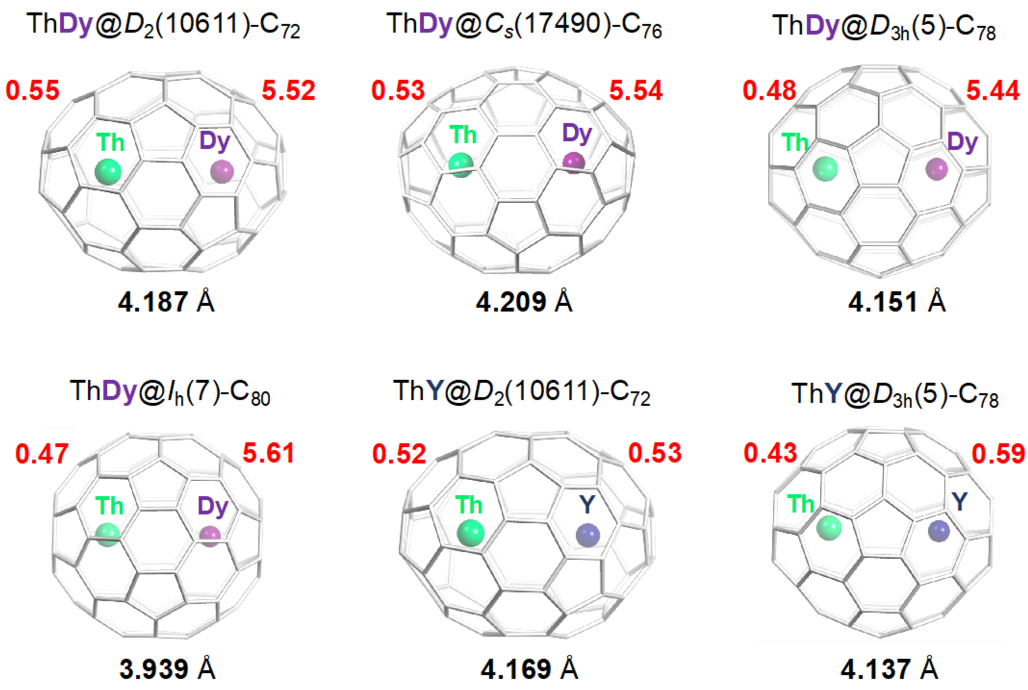

Supplementary Fig.10. DFT-optimized spin-septet ground-state (GS) geometries of ThDy@D_2(10611)-C_{72}, ThDy@C_s(17490)-C_{76}, ThDy@D_{3h}(5)-C_{78}, and ThDy@I_h(7)-C_{80}, and spin-doublet GS structures of ThY@D_2(10611)-C_{72} and ThY@D_{3h}(5)-C_{78}. Metal atoms (Th in green, Dy in purple, and Y in blue) with the corresponding atomic Mulliken spin densities (in red) and metal-metal distances (in Å) are indicated.

For ThDy@D_2(10611)-C_{72}, the metal atom Th is located in the center of a [5,5] bond with metal-cage distances of 2.529 Å. The Dy is on the opposite site, which is also located to the center of a [5,5] bond (2.409 Å). For ThDy@C_s(17490)-C_{76}, shorter metal-cage distances are found for Th (2.515 Å) and Dy (2.401 Å). For ThDy@D_{3h}(5)-C_{78}, the metal atoms reside over the C_3 axis of D_{3h}(5)-C_{78} cage and each of them is located in the center of a sumanene-type hexagon with the six M-cage distances of 2.509 Å. For ThDy@I_h(7)-C_{80}, both metals are located in the center of a hexagon, where the six Th-cage distances range between 2.502-2.528 Å, while the Dy-cage distances are between 2.418-2.437 Å.

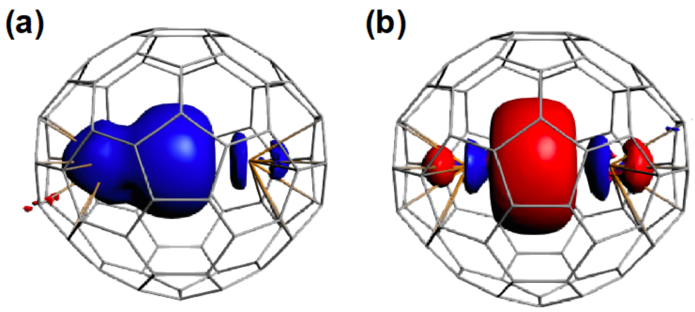

(c)

Th	Dy
$7p_z$ (18.4%)	$6p_z$ (24.3%)
7s (15.7%)	6s (17.7%)
$6d_{z2}$ (7.2%)	$5d_{z2}$ (7.5%)
$5f_{z3}$ (2.0%)	

Supplementary Fig.11. (a) Spin density distribution for ThDy@D_{3h}(5)-C_{78} shown with an isosurface of ±0.002 au. (b) Isosurface (±0.030 au) of the singly occupied metal-metal bond orbital for ThDy@D_{3h}(5)-C_{78}. (c) Molecular orbital contributions of the σ-type bonding orbital formed essentially by ns and np metal orbitals.

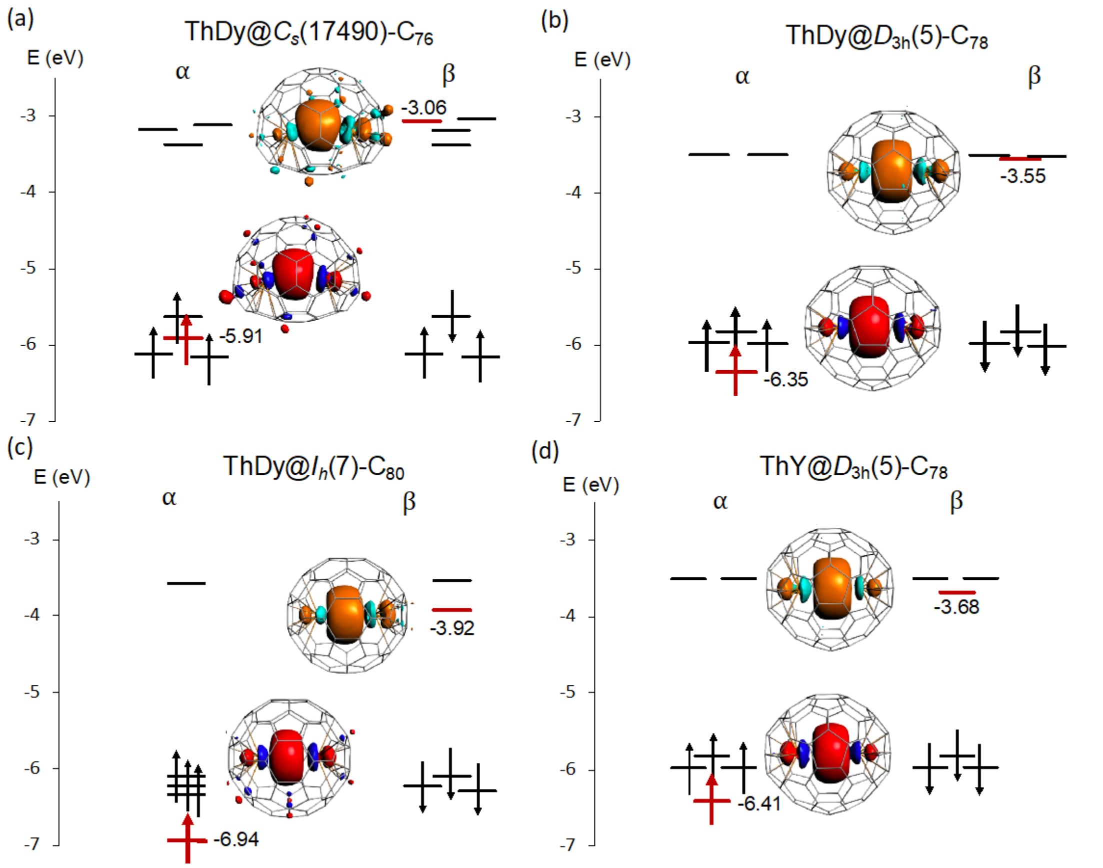

Supplementary Fig.12. Molecular orbital (MO) diagram for the ground spin-septet state of (a) ThDy@C_s(17490)-C_{76}, (b) ThDy@D_{3h}(5)-C_{78}, and (c) ThDy@I_h(7)-C_{80} and, the ground spin-doublet state of (d) ThY@D_{3h}(5)-C_{78}. Alpha (α)- and beta (β)-spin orbitals are plotted separately. Delocalized sigma orbital a_1 (α- and β-spins) levels are plotted in red with the associated MO isosurfaces (±0.030 au) shown in the side. Dy f^9 MOs are found at around ~ -12 eV.

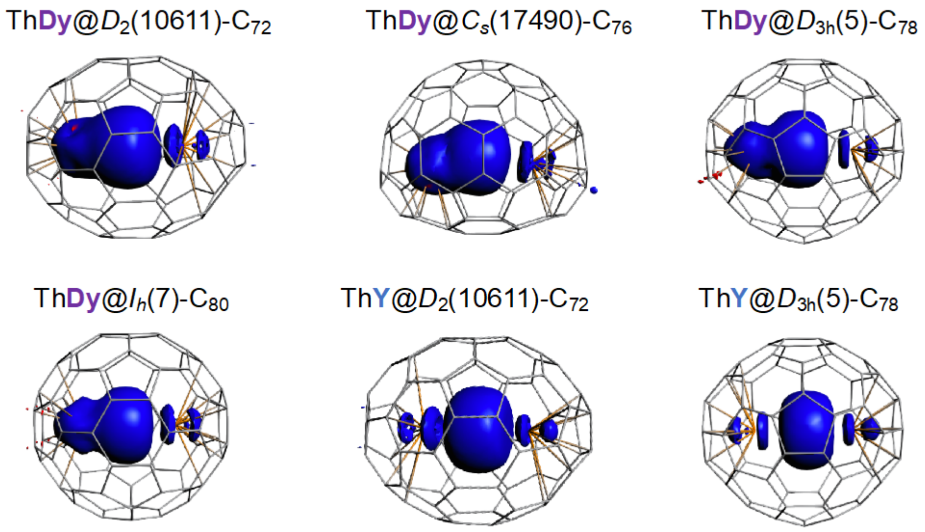

Supplementary Fig.13. Spin density distribution with an isosurface of ±0.002 au for ThDy@D_2(10611)-C_{72}, ThDy@C_s(17490)-C_{76}, ThDy@D_{3h}(5)-C_{78}, ThDy@I_h(7)-C_{80}, ThY@D_2(10611)-C_{72} and ThY@D_{3h}(5)-C_{78}.

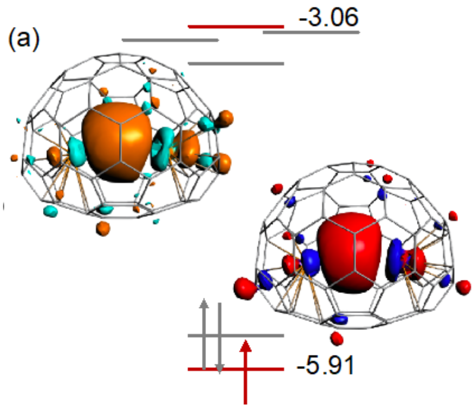

(b)

Dy	Th
$5p_x$ (15.9%)	6s (14.6%)
5s (15.8%)	$6p_x$ (8.6%)
$4d_{2-y2}$ (2.4%)	$5d_{xz}$ (6.7%)
	$6p_z$ (6.4%)
	$4f_z$ (2.0%)

Supplementary Fig.14. Electronic structure properties for ThDy@C_s(17490)-C_{76}. (a) Molecular orbital (MO) diagram. The energy levels of the delocalized sigma orbital $a1$ (for alpha-and beta spins) are drawn in red and the associated MO isosurfaces (±0.03 a.u.) with the corresponding MO energy (in eV) are shown on the side. Although a spin unrestricted calculation was performed, paired electrons are drawn to denote levels with both the alpha and beta components occupied. The energy scale corresponds to the alpha-spin levels. (b) Molecular orbital contributions of the σ-type bonding orbital formed essentially by ns and np metal orbitals.

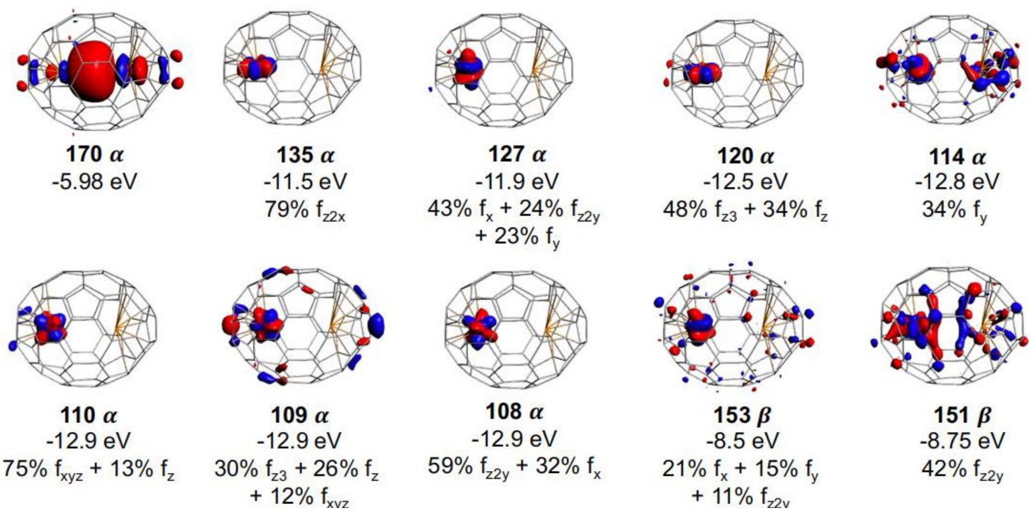

Supplementary Fig.15. Isosurface (±0.030 au) of the molecular orbitals (MOs) for the σ bonding and Dy f^9 of ThDy@D_2(10611)-C_{72}. Alpha (α)- and beta (β)-spin orbitals are plotted. Corresponding orbital energy (in eV) is indicated, as well as, the contributions (in %) of the MO.

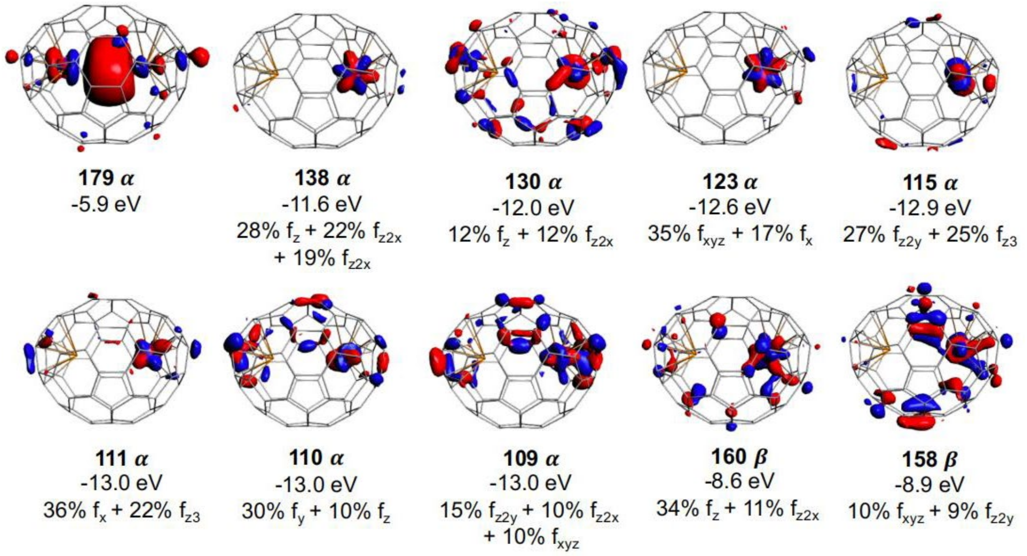

Supplementary Fig.16. Isosurface (±0.030 au) of the molecular orbitals (MOs) for the σ bonding and Dy f^9 of ThDy@C_s(17490)-C_{76}. Alpha (α)- and beta (β)-spin orbitals are plotted. Corresponding orbital energy (in eV) is indicated, as well as, the contributions (in %) of the MO.

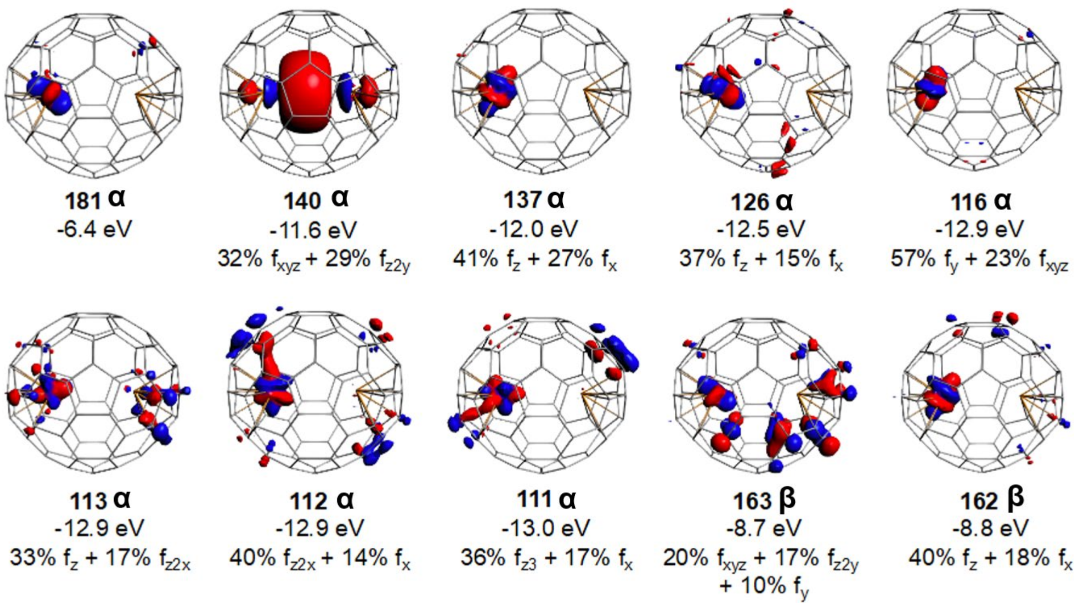

Supplementary Fig.17. Isosurface (±0.030 au) of the molecular orbitals (MOs) for the σ bonding and Dy f^9 of ThDy@D_{3h}(5)-C_{78}. Alpha (α)- and beta (β)-spin orbitals are plotted. Corresponding orbital energy (in eV) is indicated, as well as, the contributions (in %) of the MO.

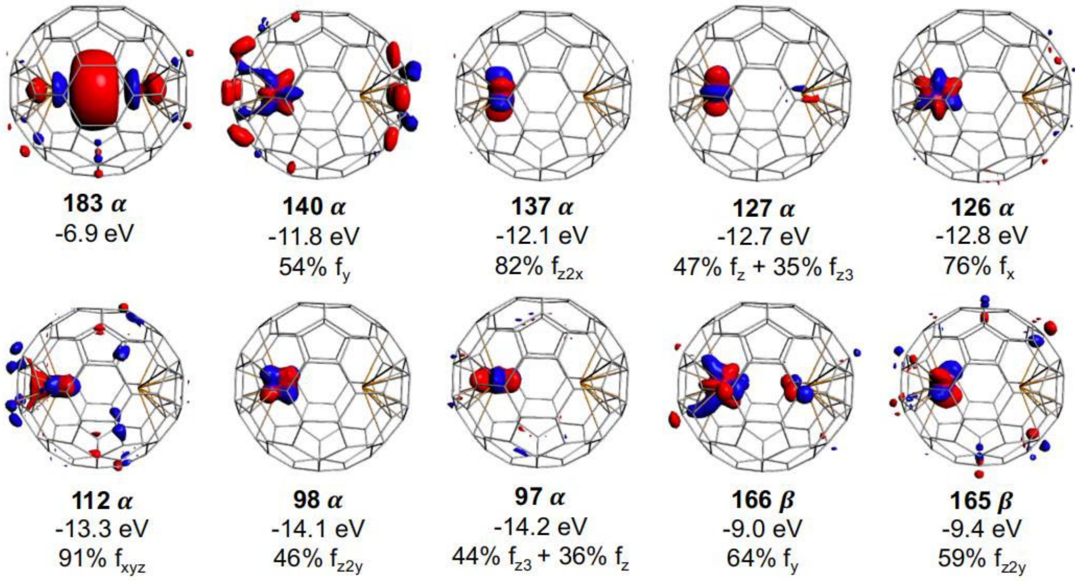

Supplementary Fig.18. Isosurface (±0.030 au) of the molecular orbitals (MOs) for the σ bonding and Dy f^9 of ThDy@I_h(7)-C_{80}. Alpha (α)- and beta (β)-spin orbitals are plotted. Corresponding orbital energy (in eV) is indicated, as well as, the contributions (in %) of the MO.

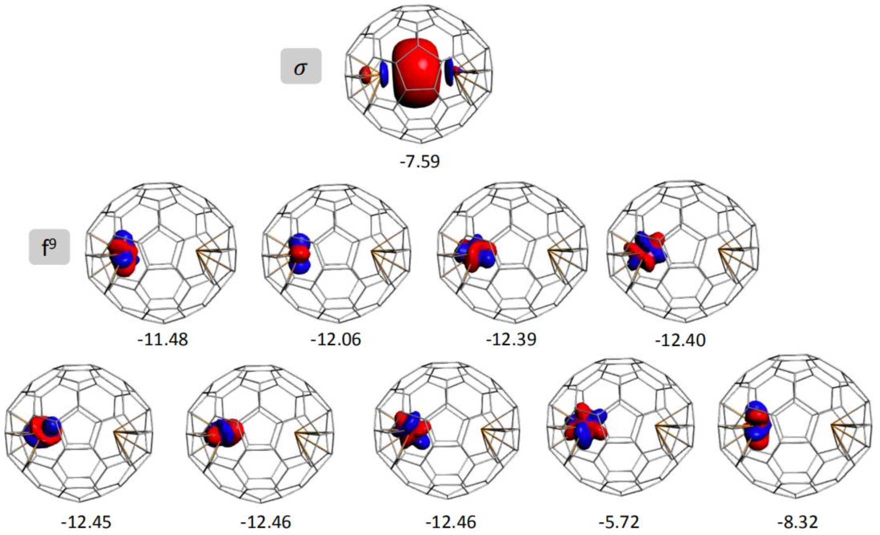

Supplementary Fig.19. Localized (Boys-Foster) molecular orbitals (LMOs) for ThDy@D_{3h}(5)-C_{78}. Isosurface (±0.03 au) of the LMOs in the ThDy moiety bonding; bonding and non-bonding f electrons of Dy. Orbital energies (in eV) are indicated for each orbital. The beta (β)-spin orbitals are the ones at -5.72 and -8.32 eV.

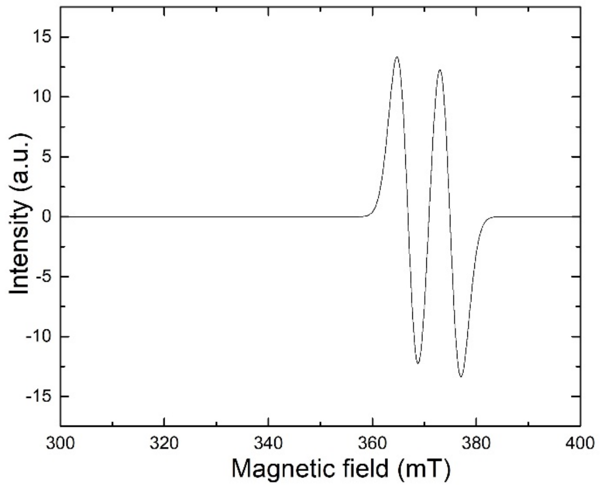

Supplementary Fig. 20. Calculated EPR spectrum of ThY@D_{3h}(5)-C_{78}.

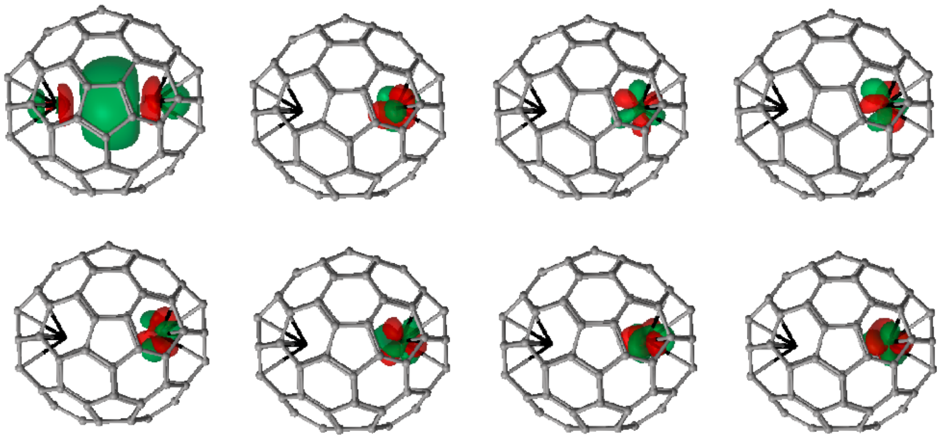

Supplementary Fig.21. Isosurface (±0.03 a.u.) of the RAS2 molecular orbitals (MOs) for the spin-septet ground state of ThDy@D_{3h}(5)-C_{78}.

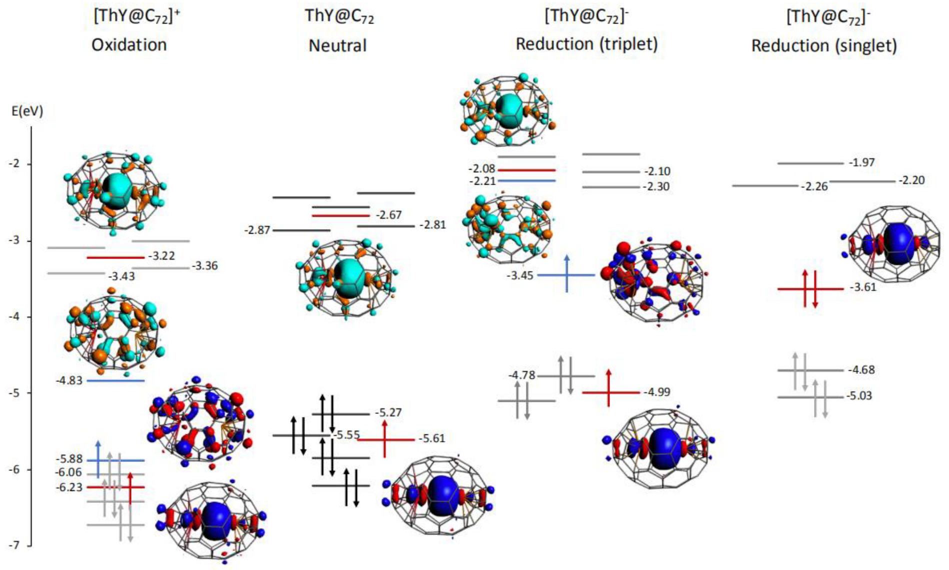

Supplementary Fig.22. Representation of the molecular orbital diagrams for the neutral, oxidation and reduced forms of ThY@D_2(10611)-C_{72}. Both singlet and triplet-spin states for the reduction are plotted. Delocalized sigma orbital *a*1 (α- and β-spins) levels are plotted in red with the associated MO isosurfaces (±0.030 au) shown in the side. Orbital energies (in eV) are indicated for each orbital.

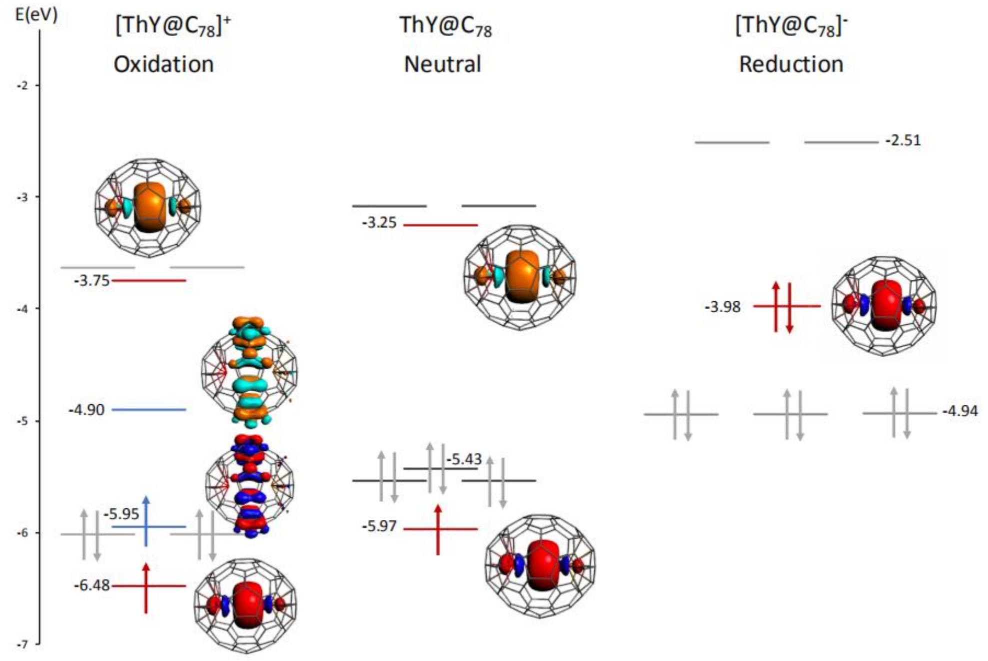

Supplementary Fig.23. Representation of the molecular orbital diagrams for the neutral, oxidation and reduced forms of ThY@D_{3h}(5)-C_{78}. Delocalized sigma orbital $a1$ (α- and β-spins) levels are plotted in red with the associated MO isosurfaces (±0.030 au) shown in the side. Orbital energies (in eV) are indicated for each orbital.

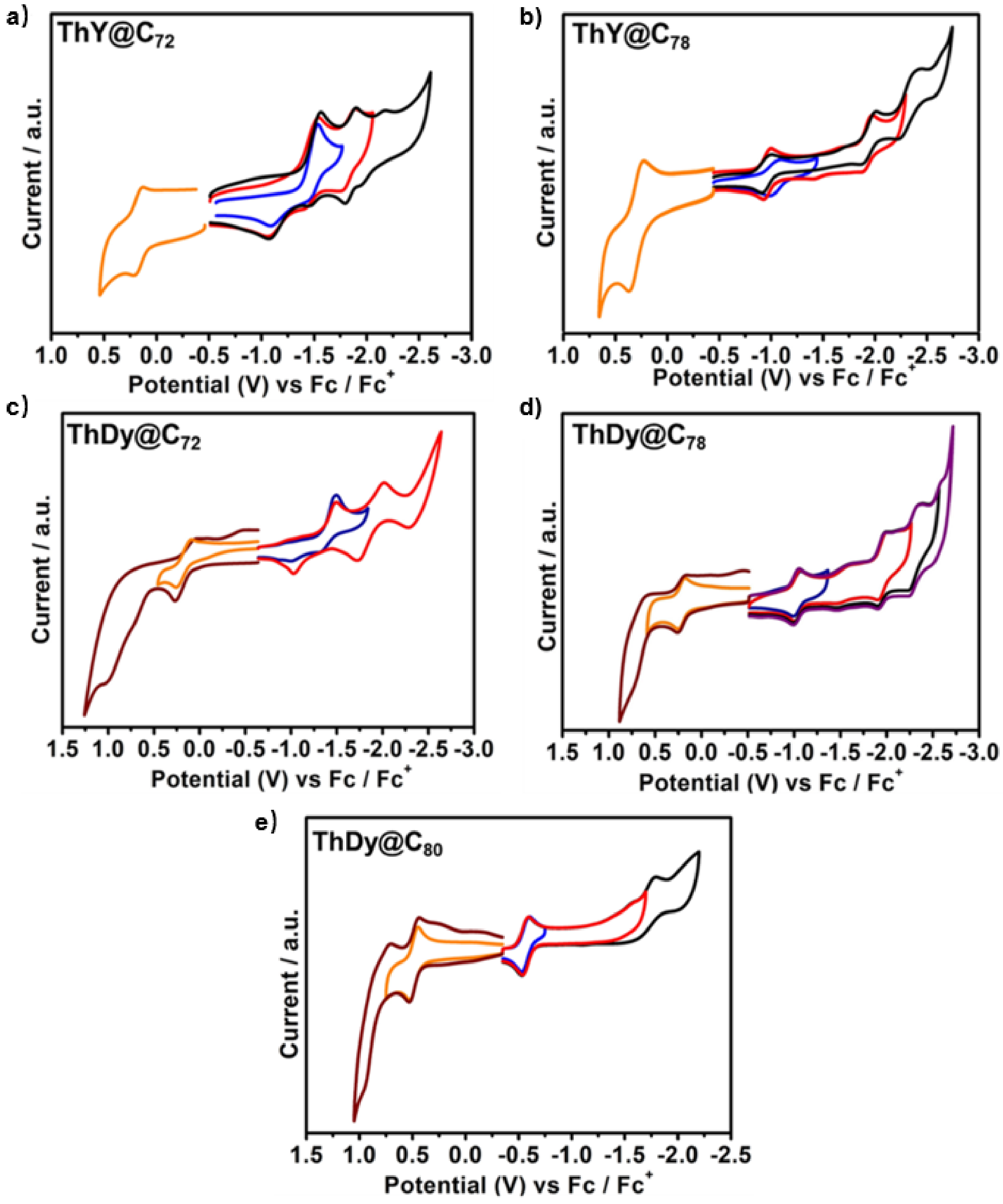

Supplementary Fig.24. Cyclic voltammogram of (a) ThY@D_2(10611)-C_{72}, (b) ThY@D_{3h}(5)-C_{78}, (c) ThDy@D_2(10611)-C_{72}, (d) ThDy@D_{3h}(5)-C_{78} and (e) ThDy@I_h(7)-C_{80} in *o*-dichlorobenzene (0.05 M (*n*-Bu)$_4$NPF$_6$; scan rate 100 mV/s for CV).

Electrochemical studies. The redox properties of ThDy@C_{2n} (2*n* = 72, 78, and 80) and ThY@C_{2n} (2*n* = 72 and 78) were investigated by cyclic voltammetry (CV). Overall, for ThDy@C_{2n} (2*n* = 72, 78, and 80), both the first reduction potentials and oxidation potentials positively shift from C_{72} to C_{80}, with the increasing size of the fullerene cage. On the other hand, their electrochemical gaps decrease from 1.67 eV for ThDy@D_2(10611)-C_{72} to 1.14 eV for ThDy@I_h(7)-C_{80}. Notably, the first reduction potential (-1.49 V) of ThDy@D_2(10611)-C_{72} is dramatically negatively shifted compared to the corresponding values of the La_2@D_2(10611)-C_{72} (-0.68 V) and Ce_2@D_2(10611)-C_{72} (-0.81 V) while the latter two present very similar redox potentials. The same phenomena can be observed for ThDy@D_{3h}(5)-C_{78} when compared to its lanthanide analog La_2@D_{3h}(5)-C_{78}. Their first reduction potential shifts from -0.40 eV for La_2@D_{3h}(5)-C_{78} to -1.03 eV for ThDy@D_{3h}(5)-C_{78}. For ThDy@I_h(7)-C_{80}, this trend continues, but the difference between ThDy@I_h(7)-C_{80} and its lanthanide analogue La_2@I_h(7)-C_{80} becomes smaller.

The first reduction process of these mixed An-Ln endofullerenes, is predicted to take place at the Th-Y sigma orbital, which is the HOMO in the singlet spin configuration of the reduced system of ThY@C_{78} (see Supplementary Fig. 22 and 23 and Supplementary Table 11). However, for ThY@C_{72}, the reduction to triplet state with the additional electron delocalized over the fullerene is 1.1 $kcal \cdot mol^{-1}$ lower than the singlet state. Given the MO diagram of Fig. 3 and that at DFT level the stability of the singlet state is somewhat underestimated, we predict that the first reduction for all ThX@C_{2n} (2*n* = 72, 78, and 80; X = Y and Dy) EMFs reported in this article occur at the sigma Th-Ln orbital.

Supplementary Fig.25 presents the UV-vis−NIR absorption spectra of ThDy@C_{2n} ($2n$ = 72, 76, 78, and 80) and ThY@C_{2n} ($2n$ = 72 and 78). The absorption features of ThDy@C_{2n} ($2n$ = 72, 76, 78, and 80) vary dramatically with the cage size and symmetry. As shown in Supplementary Fig.25, one salient sharp (568 nm) and two weak (439 and 702 nm) absorption peaks were observed in ThDy@C_{72}. Similar peaks were found in the absorption spectra of M_2@D_2(10611)-C_{72} (M = La and Pr),[1, 2] which indicates that ThDy@C_{72} processes the same D_2(10611) cage. Likewise, the absorption curve of ThDy@C_{76}, which shows minor peaks at 638 and 750 nm, is similar to those of La_2@C_s(17490)-C_{76}[3] (613 and 698 nm) and the cluster metallofullerene $DySc_2N$@C_s(17490)-C_{76} (586 and 687 nm),[4] indicating the same cage symmetry. Further, the similar peak absorption indicates that ThDy@C_{78} shares resembling metal-cage interactions to La_2@D_{3h}(5)-C_{78} as well as six-electrons transfer pattern.[5] The spectrum of ThDy@C_{80} is rather featureless except for minor absorption peaks observed at 500 nm, similar to those of the Th_2@I_h(7)-C_{80} and U_2@I_h(7)-C_{80},[6, 7] typical for I_h(7)-C_{80} cage based EMFs. Furthermore, these six Thorium-based di-EMFs possess relatively large optical gaps, ranging from 1.04 to 1.28 eV (Table S1), suggesting their relatively high thermodynamic stabilities.

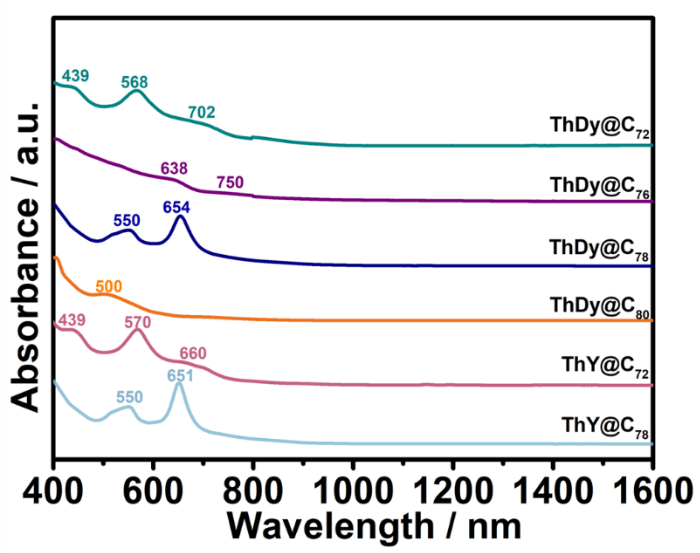

Supplementary Fig.25. UV-vis−NIR absorption spectra of purified ThDy@C_{2n} ($2n$ = 72, 76, 78, and 80) and ThY@C_{2n} ($2n$ = 72 and 78) dissolved in CS_2.

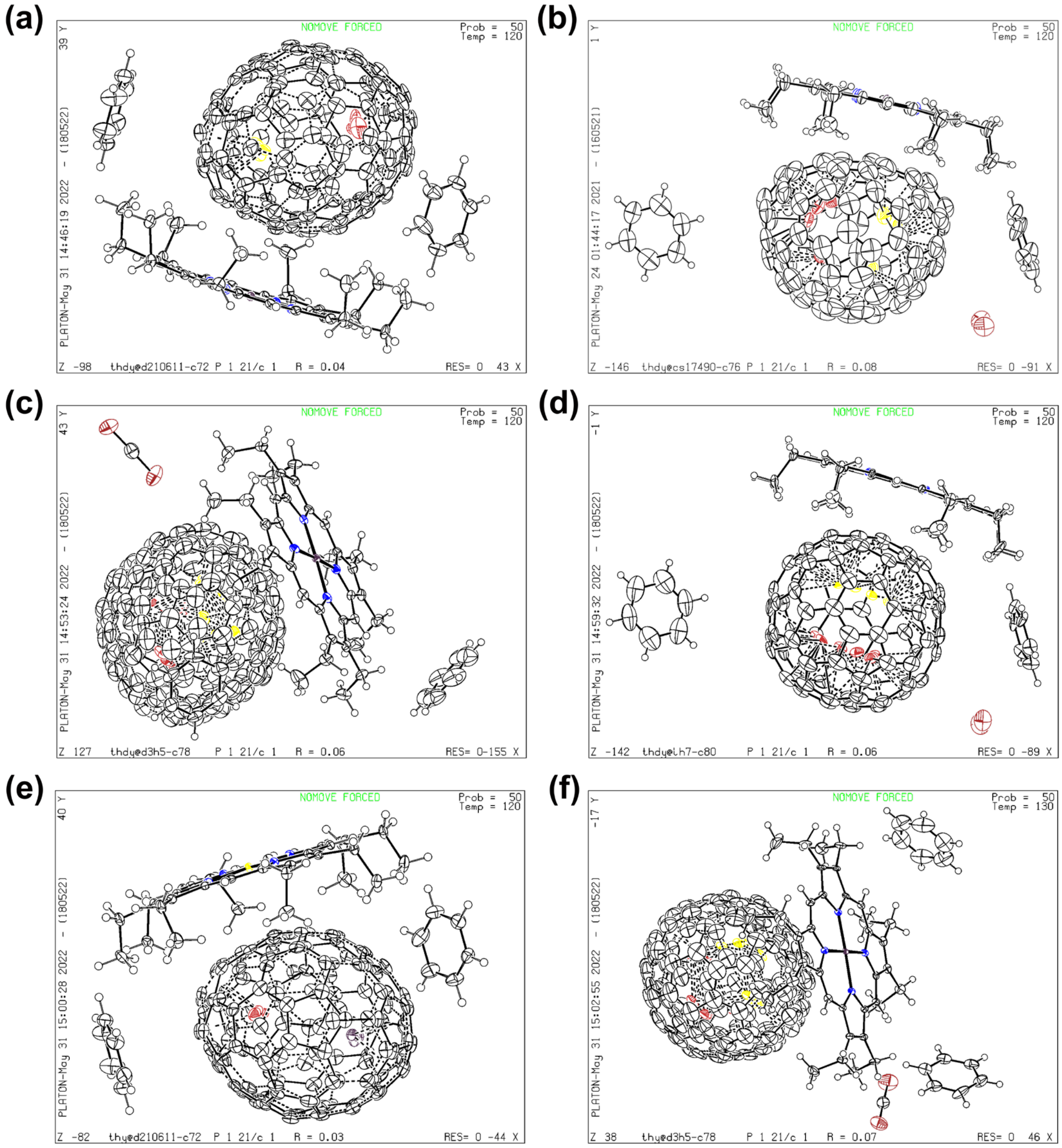

Supplementary Fig.26. ORTER-style illustration with probability ellipsoids for (a) ThDy@D_2(10611)-C_{72} (CCDC-2108663), (b) ThDy@C_s(17490)-C_{76} (CCDC-2108664), (c) ThDy@D_{3h}(5)-C_{78} (CCDC-2108688), (d) ThDy@I_h(7)-C_{80} (CCDC-2108689), (e) ThY@D_2(10611)-C_{72} (CCDC-2108690), and (f) ThY@D_{3h}(5)-C_{78} (CCDC-2108694).

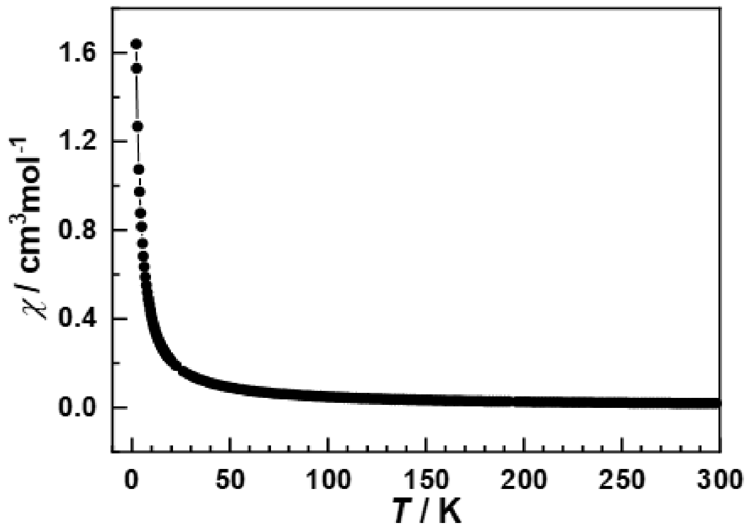

Supplementary Fig.27. Experimental temperature dependence of χ for ThDy@D_{3h}(5)-C_{78}

Supplementary Table 1. The cage disorder and the shortest Ni-to-Cage distance of ThX@C_{2n}·[Ni^{II}(OEP)] (X = Dy and Y, 2n = 72, 76, 78, and 80).

EMFs	Cage orientation<br>Cage A : Cage B	Ni-to-cage A distance/Å
ThDy@D_2(10611)-C_{72}	0.55:0.45	Ni-C21A, 2.804 Å
ThDy@C_s(17490)-C_{76}	one orientation	Ni-C18A, 2.937 Å
ThDy@D_{3h}(5)-C_{78}	0.51:0.49	Ni-C14A, 2.856 Å
ThDy@I_h(7)-C_{80}	one orientation	Ni-C11A, 2.769 Å
ThY@D_2(10611)-C_{72}	0.53:0.47	Ni-C52A, 2.773 Å
ThY@D_{3h}(5)-C_{78}	0.51:0.49	Ni-C12A, 2.866 Å

Supplementary Table 2. The fractional occupancies of the metal positions in ThDy@C_{2n} (2n = 72, 76, 78, and 80) and ThY@C_{2n} (2n = 72 and 78).

EMFs	Fractional metal ions of Th, Dy or Y									
ThDy@D_2(10611)-C_{72}	Th1	Th2	Th3							
	0.842(2)	0.103(2)	0.0546(16)							
	Dy1	Dy2	Dy3							
	0.842(2)	0.103(2)	0.0546(16)							
ThDy@C_s(17490)-C_{76}	Th1	Th2	Th3	Th4						
	0.670(3)	0.179(3)	0.1110(14)	0.0402(14)						
	Dy1	Dy2	Dy3	Dy4						
	0.659(2)	0.155(2)	0.1602(18)	0.0259(16)						
ThDy@D_{3h}(5)-C_{78}	Th1	Th2	Th3	Th4	Th5	Th6	Th7	Th8	Th9	Th10
	0.581(2)	0.051(2)	0.0514(17)	0.0719(16)	0.0137(13)	0.0318(8)	0.0403(9)	0.0539(10)	0.0941(12)	0.0111(7)
	Dy1	Dy2	Dy3	Dy4	Dy5	Dy6	Dy7	Dy8		
	0.589(2)	0.0593(19)	0.080(2)	0.057(2)	0.0352(13)	0.0400(12)	0.0533(14)	0.0859(15)		
ThDy@I_h(7)-C_{80}	Th1	Th2	Th3	Th4	Th5	Th6	Th7			
	0.7221(19)	0.0193(12)	0.0125(8)	0.0204(9)	0.1861(13)	0.0151(8)	0.0245(9)			
	Dy1	Dy2	Dy3	Dy4	Dy5	Dy6				
	0.7184(18)	0.0247(12)	0.0212(13)	0.1982(17)	0.0151(11)	0.0224(12)				
ThY@D_2(10611)-C_{72}	Th1	Th2	Th3							
	0.9281(13)	0.0555(12)	0.0160(4)							
	Y1	Y2	Y3							
	0.9281(13)	0.0555(12)	0.0160(4)							
ThY@D_{3h}(5)-C_{78}	Th1	Th2	Th3	Th4	Th5	Th6	Th7			
	0.63	0.07	0.1	0.025	0.05	0.05	0.075			
	Y1	Y2	Y3	Y4	Y5	Y6	Y7			
	0.63	0.07	0.1	0.025	0.05	0.05	0.075			

Supplementary Table 3. Closest metal-to-cage contacts in ThDy@D_2(10611)-C_{72}, ThDy@C_s(17490)-C_{76}, ThDy@D_{3h}(5)-C_{78} and ThDy@I_h(7)-C_{80}.

Compounds	The shortest metal-cage	Bond length / Å	Range / Å
ThDy@D_2(10611)-C_{72}	Th1-C1A	2.475	2.475-2.605
	Th1-C2A	2.512	
	Th1-C3A	2.605	
	Th1-C4A	2.601	
	Th1-C5A	2.524	
	Th1-C6A	2.573	
	Th1-C7A	2.537	
	Th1-C8A	2.476	
	Dy1-64A	2.551	2.404-2.551
	Dy1-65A	2.548	
	Dy1-66A	2.428	
	Dy1-67A	2.404	
	Dy1-68A	2.435	
	Dy1-70A	2.496	
	Dy1-71A	2.464	
	Dy1-72A	2.441	
ThDy@C_s(17490)-C_{76}	Th1-C1	2.433	2.433-2.448
	Th1-C2	2.435	
	Th1-C8	2.448	
	Dy1-C71	2.312	2.312-2.354
	Dy1-C72	2.324	
	Dy1-C73	2.354	
ThDy@D_{3h}(5)-C_{78}	Th1-C1A	2.451	2.355-2.477
	Th1-C2A	2.403	
	Th1-C3A	2.355	
	Th1-C4A	2.437	
	Th1-C5A	2.461	
	Th1-C6A	2.477	
	Dy1-67A	2.351	2.276-2.423
	Dy1-68A	2.383	
	Dy1-72A	2.423	
	Dy1-73A	2.313	
	Dy1-77A	2.276	
	Dy1-78A	2.283	
ThDy@I_h(7)-C_{80}	Th1-C1A	2.466	2.452-2.504
	Th1-C2A	2.457	
	Th1-C3A	2.492	
	Th1-C4A	2.504	
	Th1-C5A	2.452	

	Th1-C6A	2.493	
	Dy1-68A	2.422	2.385-2.440
	Dy1-69A	2.420	
	Dy1-73A	2.385	
	Dy1-74A	2.440	
	Dy1-76A	2.394	
	Dy1-80A	2.396	

Supplementary Table 4. Closest metal-to-cage contacts in ThY@D_2(10611)-C_{72} and ThY@D_{3h}(5)-C_{78}.

Compounds	The shortest metal-cage	Bond length / Å	Range / Å
ThY@D_2(10611)-C_{72}	Th1-63A	2.607	2.489-2.611
	Th1-64A	2.611	
	Th1-66A	2.537	
	Th1-67A	2.556	
	Th1-68A	2.537	
	Th1-69A	2.494	
	Th1-71A	2.489	
	Th1-72A	2.513	
	Y1-C1A	2.392	2.392-2.549
	Y1-C2A	2.430	
	Y1-C3A	2.480	
	Y1-C4A	2.468	
	Y1-C5A	2.442	
	Y1-C6A	2.539	
	Y1-C7A	2.549	
	Y1-C8A	2.451	
ThY@D_{3h}(5)-C_{78}	Th1-C1A	2.477	2.376-2.454
	Th1-C2A	2.376	
	Th1-C3A	2.418	
	Th1-C4A	2.454	
	Th1-C5A	2.495	
	Th1-C6A	2.477	
	Y1-C67A	2.361	2.273-2.385
	Y1-C68A	2.385	
	Y1-C72A	2.349	
	Y1-C73A	2.310	
	Y1-C77A	2.273	
	Y1-C78A	2.275	

Supplementary Table 5. Crystallographic data of ThDy@D_2(10611)-C_{72}/Ni^{II}(OEP), ThDy@C_s(17490)-C_{76}/Ni^{II}(OEP), ThDy@D_{3h}(5)-C_{78}/Ni^{II}(OEP) and ThDy@I_h(7)-C_{80}/Ni^{II}(OEP).

Compound	ThDy@D_2(10611)-C_{72}/Ni^{II}(OEP)	ThDy@C_s(17490)-C_{76}/Ni^{II}(OEP)	ThDy@D_{3h}(5)-C_{78}/Ni^{II}(OEP)	ThDy@I_h(7)-C_{80}/Ni^{II}(OEP)
Chemical formula	$C_{120}H_{56}DyN_4NiTh$	$C_{122}H_{53}DyN_4NiS_2Th$	$C_{124}H_{53}DyN_4NiS_2Th$	$C_{126}H_{53}DyN_4NiS_2Th$
Formula weight	2006.93	2092.05	2116.07	2140.09
Crystal colour	black	black	black	black
Crystal system	monoclinic	monoclinic	monoclinic	monoclinic
Space group	$P2_1/c$	$P2_1/c$	$P2_1/c$	$P2_1/c$
a, Å	19.0405(9)	17.7398(9)	17.6245(13)	17.6074(13)
b, Å	14.6490(7)	16.7164(10)	16.8820(11)	17.0153(12)
c, Å	25.4436(12)	26.5569(15)	26.528(2)	26.800(2)
α, deg	90	90	90	90
β, deg	92.969(2)	109.436(2)	106.699(4)	106.809(4)
γ, deg	90	90	90	90
V, $Å^3$	7087.3(6)	7426.5(7)	7560.1(9)	7686.0(10)
Z	4	4	4	4
T/K	120	120	120	120
Radiation Source	Ga $K_α$	Ga $K_α$	Ga $K_α$	Ga $K_α$
λ, Å	1.34	1.34	1.34	1.34
μ, mm^{-1}	11.736	11.555	11.360	11.181
R1 (I > 2.0 σ(I)	0.0449	0.0828	0.0759	0.0638
wR2(all data)	0.1190	0.2457	0.1804	0.1628
GOF	1.031	1.023	1.026	1.024

Supplementary Table 6. Crystallographic data of ThY@D_2(10611)-C_{72}/Ni^{II}(OEP) and ThY@D_{3h}(5)-C_{78}/Ni^{II}(OEP).

Chemical formula	ThY@D_2(10611)-C_{72}/Ni^{II}(OEP)	ThY@D_{3h}(5)-C_{78}/Ni^{II}(OEP)
Formula weight	$C_{120}H_{56}N_4NiThY$	$C_{124}H_{53}N_4NiS_2ThY$
Crystal colour	1933.34	2042.48
Crystal system	monoclinic	monoclinic
Space group	$P2_1$/c	$P2_1$/c
a, Å	18.9950(13)	17.627(9)
b, Å	14.5855(9)	16.875(6)
c, Å	25.4450(16)	26.519(12)
α, deg	90	90
β, deg	92.812(3)	106.68(3)
γ, deg	90	90
V, $Å^3$	7041.1(8)	7556(6)
Z	4	4
T/K	120	130
Radiation Source	Ga $K_α$	Cu $K_α$
λ, Å	1.34	1.54
μ, mm^{-1}	7.028	8.622
R1 (I > 2.0 σ(I)	0.0266	0.0690
wR2(all data)	0.0654	0.1966
GOF	1.026	1.068

Supplementary Table 7. Relative energies (in kcal·mol^{-1}) of the RASSCF calculations for the different spin states, and lowest-energy states for ThDy@D_{3h}(5)-C_{78}.

Spin	State	RASSCF Erel
Quintet	1	11.0
	2	11.3
	3	11.3
	4	11.4
	5	11.6
	6	11.6
	7	11.7
	8	11.7
	9	11.9
	10	11.9
	11	11.9
	12	32.6
Septet	1	0.0
	2	0.0
	3	0.6
	4	0.8
	5	0.8
	6	1.0
	7	1.2
	8	1.2
	9	1.3
	10	1.3
	11	1.5
	12	22.1

Supplementary Table 8. Relative energies (in kcal·mol^{-1}) and spin distributions of active orbitals with the corresponding weight of the dominant electronic configuration (in %) for the lowest states determined from CASSCF calculations for ThDy@D_{3h}(5)-C_{78}. The spin coupling of open shells is indicated as u (spin up) and d (spin down), 2 means doubly occupied.

Spin	State	Erel	Occupation	Weigth
	1	11.0	22uuuuud	17
Quintet	2	11.3	u22uuuud	28
	3	11.3	u2uu2uud	21
	1	0.0	2uu2uuuu	31
	2	0.0	2uuuuu2u	17
	3	0.6	22uuuuuu	23
Septet	4	0.8	u22uuuuu	29
	5	0.8	2uuuu2uu	22
	6	1.0	uu22uuuu	25

Supplementary Table 9. Redox potentials (V vs. Fc/Fc$^+$) and electrochemical band gaps of ThY@D_2(10611)-C_{72}, ThY@D_{3h}(5)-C_{78}, ThDy@D_2(10611)-C_{72}, ThDy@D_{3h}(5)-C_{78}, ThDy@I_h(7)-C_{80} and the selected reference EMFs.

	$E^{2+/+}$	$E^{+/0}$	$E^{0/-}$	$E^{-/2-}$	$E^{2-/3-}$	$E^{3-/4-}$	E_{gap}(V)	ref
ThDy@D_2(10611)-C_{72}	1.08^b	0.18^a	-1.49^b	-1.88^a			1.67	this work
ThY@D_2(10611)-C_{72}		0.16^a	-1.52^b	-1.84^b	-2.17^b		1.68	this work
La_2@D_2(10611)-C_{72}	0.75^b	0.24^a	-0.68^b	-1.92^b			0.92	8
Ce_2@D_2(10611)-C_{72}	0.82^b	0.18^a	-0.81^b	-1.86^a			0.99	9
ThDy@D_{3h}(5)-C_{78}	0.73^b	0.22^a	-1.03^a	-1.95^a	-2.35^b	-2.55^a	1.25	this work
ThY@D_{3h}(5)-C_{78}	0.98^b	0.31^a	-0.97^a	-1.96^a	-2.41^a		1.28	this work
La_2@D_{3h}(5)-C_{78}	0.62^a	0.26^a	-0.40^a	-1.84^a	-2.28^a		0.66	5
ThDy@I_h(7)-C_{80}	0.96^b	0.49^a	-0.66^a	-1.58^b	-1.79^b		1.14	this work
U_2@I_h(7)-C_{80}	1.16^b	0.40^a	-0.44^a	-1.58^b	-1.78^b	-2.28^b	0.91	7
La_2@I_h(7)-C_{80}	0.95^b	0.56^b	-0.31^a	-1.71^a	-2.13^b		0.87	10

Supplementary Table 10. Experimental and calculated first oxidation and reduction potentials (in eV) for ThY@C_{2n} ($2n$ = 72 and 78).

	**Ox1**	**Red1**
**ThY@C_{72}**		
Calculation	0.29	-1.62
Experimental	0.16	-1.52
**ThY@C_{78}**		
Calculation	0.42	-1.21
Experimental	0.31	-0.97

Supplementary Table 11. Relative energies (in kcal·mol^{-1}) for the singlet and triplet spin states for the oxidation and reduction of ThY@C_{2n} ($2n$ = 72 and 78).

		ThY@C_{72}	ThY@C_{78}
**Oxidation**	Singlet	4.1	9.9
	Triplet	0.0	0.0
**Reduction**	Singlet	1.1	0.0
	Triplet	0.0	5.1

Supplementary Table 12. The HPLC retention time and details of the UV-vis-NIR absorptions of ThDy@C_{2n} ($2n$ = 72, 76, 78, and 80) and ThY@C_{2n} ($2n$ = 72 and 78).

Compound	HPLC Retention time (min)	Vis-NIR absorb bands (nm)	Oneset (nm)	Optical bandgap (eV)
ThDy@C_{72}	36-42	568, 702	1053	1.18
ThDy@C_{76}	66-74	638, 750	1195	1.04
ThDy@C_{78}	42-48	550, 654	1040	1.19
ThDy@C_{80}	74-82	500, 689	970	1.28
ThY@C_{72}	34-40	570, 680, 695	1005	1.23
ThY@C_{78}	42-48	550, 651	1025	1.21

[a] HPLC conditions: Buckyprep column, 4 mL/min.

[b] Optical bandgap (eV) = 1240/onset (nm).

Supplementary Table 13: Selected distances compared between ThDy@D_{3h}(5)-C_{78} and Dy_2@C_{79}N/ Dy_2@C_{80}(CH_2Ph).

Compounds	Dy-to-ring-centroid distance (Å)			Dy-metal distance (Å)	△d* (Å)	
	Dy1	Dy2	average		Dy-to-ring-centroid distance	Dy-metal distance
ThDy@D_{3h}(5)-C_{78}	1.867			4.136		
Dy_2@C_{79}N[11]	1.893	1.939	1.916	3.890	-0.019	0.246
Dy_2@C_{80}(CH_2Ph)[12]	1.987	1.988	1.988	3.896	-0.121	0.240

*the difference of distances between ThDy@D_{3h}(5)-C_{78} and other compound.

References

1. Zhao, Y.-L., Yu, H.-T. & Lian, Y.-F. Experimental and theoretical evaluation of structures of $Pr_2@C_{72}$ and its functionalized adduct with adamantylidene carbene. *RSC Adv.* **6**, 115113-115119 (2016).
2. Lu, X., *et al.* Bis-Carbene Adducts of Non-IPR $La_2@C_{72}$: Localization of High Reactivity around Fused Pentagons and Electrochemical Properties. *Angew. Chem.Int. Ed.* **47**, 8642-8645 (2008).
3. Suzuki, M., *et al.* $La_2@C_s(17490)\text{-}C_{76}$: A new non-IPR dimetallic metallofullerene featuring unexpectedly weak metal–pentalene interactions. *Chem. Eur. J.* **19**, 17125-17130 (2013).
4. Yang, S., Popov, A.A. & Dunsch, L. The role of an asymmetric nitride cluster on a Fullerene Cage: the Non-IPR endohedral $DySc_2N@C_{76}$. *J. Phys. Chem. B* **111**, 13659-13663 (2007).
5. Cao, B.P., *et al.* Isolation, characterization, and theoretical study of $La_2@C_{78}$. *J. Am. Chem. Soc.* **126**, 9164-9165 (2004).
6. Zhuang, J.M.-M., R. Zhang, J. Wang, Y. Yao, Y. R. & Pei, C.R.-F., A. Wang, S. Echegoyen, L. De Graaf, C. Poblet, J. M. Chen, N. Characterization of a strong covalent $Th^{3+}\text{-}Th^{3+}$ bond inside an $I_h(7)\text{-}C_{80}$ fullerene cage. *Nat. Commun.* **12**, 2372 (2021).
7. Zhang, X., *et al.* $U_2@I_h(7)\text{-}C_{80}$: crystallographic characterization of a long-sought dimetallic actinide endohedral fullerene. *J. Am. Chem. Soc.* **140**, 3907-3915 (2018).
8. Lu, X.N., H. Tsuchiya, T. Maeda, Y. Ishitsuka, M. O. Akasaka, T. & Toki, M.S., H. Slanina, Z. Mizorogi, N. Nagase, S. Bis-carbene adducts of non-IPR $La_2@C_{72}$: localization of high reactivity around fused pentagons and electrochemical properties. *Angew. Chem. Int. Ed.* **47**, 8642-8645 (2008).
9. Yamada, M., *et al.* Spectroscopic and Theoretical Study of Endohedral Dimetallofullerene Having a Non-IPR Fullerene Cage: $Ce_2@C_{72}$. *J. Phys. Chem. A* **112**, 7627-7631 (2008).
10. Suzuki, T., *et al.* Electrochemistry and Ab Initio study of the dimetallofullerene $La_2@C_{80}$. *Angew. Chem. Int. Ed.* **34**, 1094-1096 (1995).
11. Wang, Y., *et al.* $Dy_2@C_{79}N$: a new member of dimetalloazafullerenes with strong single molecular magnetism. *Nanoscale* **12**, 11130-11135 (2020).
12. Liu, F., *et al.* Single molecule magnet with an unpaired electron trapped between two lanthanide ions inside a fullerene. *Nat. Commun.* **8**, 16098 (2017).
