# Supplementary material for: Actinide-lanthanide single electron metal-metal bond formed in mixed-valence di-metallofullerenes
Source: Nat Commun. 2023 Oct 20;14:6637. doi: 10.1038/s41467-023-42165-x (PMC10589252; doi:10.1038/s41467-023-42165-x)
Supplement: Supplementary file 3 — Description of Additional Supplementary Files [file 41467_2023_42165_MOESM3_ESM.pdf]

Description of Additional Supplementary Files

File Name: Supplementary Data 1
Description: Cif files
